# Sporulation in *Ashbya gossypii*

**DOI:** 10.3390/jof6030157

**Published:** 2020-08-29

**Authors:** Jürgen Wendland

**Affiliations:** Department of Microbiology and Biochemistry, Hochschule Geisenheim University, Von-Lade-Strasse 1, D-65366 Geisenheim, Germany; juergen.wendland@hs-gm.de; Tel.: +49-6722-502-332

**Keywords:** homothallism, pheromone signal transduction cascade, mating type, meiosis, germination, ascus, functional analysis, RNAseq

## Abstract

*Ashbya gossypii* is a filamentous ascomycete belonging to the yeast family of *Saccharomycetaceae*. At the end of its growth phase *Ashbya* generates abundant amounts of riboflavin and spores that form within sporangia derived from fragmented cellular compartments of hyphae. The length of spores differs within species of the genus. Needle-shaped *Ashbya* spores aggregate via terminal filaments. *A. gossypii* is a homothallic fungus which may possess **a** and α mating types. However, the solo-*MAT**a*** type strain is self-fertile and sporulates abundantly apparently without the need of prior mating. The central components required for the regulation of sporulation, encoded by *IME1, IME2, IME4, KAR4*, are conserved with *Saccharomyces cerevisiae*. Nutrient depletion generates a strong positive signal for sporulation via the cAMP-PKA pathway and *SOK2*, which is also essential for sporulation. Strong inhibitors of sporulation besides mutations in the central regulatory genes are the addition of exogenous cAMP or the overexpression of the mating type gene *MAT*α2. Sporulation has been dissected using gene-function analyses and global RNA-seq transcriptomics. This revealed a role of Msn2/4, another potential PKA-target, for spore wall formation and a key dual role of the protein A kinase Tpk2 at the onset of sporulation as well as for breaking the dormancy of spores to initiate germination. Recent work has provided an overview of ascus development, regulation of sporulation and spore maturation. This will be summarized in the current review with a focus on the central regulatory genes. Current research and open questions will also be discussed.

## 1. Introduction

As a plant pathogen *A. gossypii* causes a yeast spot disease termed *stigmatomycosis* e.g., on cotton, pistachio or soybean [1]. *Ashbya* is dependent on insect vectors belonging to stinkbug families (*Coreidae*, *Pentatomidae*). Thus, fungicide treatment is an effective pest-control management system. There is a more than one hundred yearlong history of *Ashbya* research starting out with characterizing *Ashbya* as a plant pathogen. Soon after its potential for riboflavin overproduction was recognized, which led to the establishment of *Ashbya* as a platform producer of vitamin B_2_ on an industrial scale [2,3,4]. Such a platform strain has expanded uses e.g., in the production of other vitamins, flavor compounds or lipids [5,6,7,8,9]. Metabolic engineering and metabolic flux studies in *Ashbya* have been used to determine and improve key enzymatic reactions in pathways required for riboflavin or gamma-lactone production [9]. *Ashbya* requires sucking insects to form plant infection as it does not produce penetration pegs or lytic enzymes. On the other hand, this makes *Ashbya* a suitable host for recombinant protein production [10].

Molecular genetic work was initiated during the early 1990s by Peter Philippsen and his group who introduced replicative and integrative transformation. This work also revealed *Ashbya’s* highly efficient homologous recombination system [11,12,13]. The tool-box for *Ashbya* is quite versatile with different marker genes, regulatable promoters, Cre-lox based marker removal and it received recent additions with CRISPR/Cas9 and CRISPR-Cpf1 for multiplex genome editing [14,15,16,17,18]. The Philippsen group then went on to establish the full genome sequence of *A. gossypii*, which provided comparative genomic proof of the Whole Genome Duplication in the *Saccharomyces* lineage [19,20,21]. Comparative genomics studies within the genus of *Eremothecium* revealed chromosome number reductions from eight to seven chromosomes in *Ashbya gossypii* and *A. aceri* and from eight to six chromosomes in *E. coryli.* The genome of *E. cymbalariae* in contrast hosts the ancestral number of eight chromosomes [19,21,22,23]. 

*Ashbya* has served as a tractable filamentous fungus to study developmental cell biology [24]. Polarized hyphal growth was found to depend on a cascade of Rho-proteins and their effectors Spa2, Bni1, Bnr1/2, Boi1/2 [24,25,26,27,28,29,30,31]. The poly-Q-protein Whi3 has a key role for the localization of mRNAs of key polarity factors Bni1 and Spa2 at sites of polarized growth [32]. This control of aggregates in a cytoplasmic space is dependent on differential phosphorylation of Whi3 [33].

Key components regulating exocytosis and endocytosis were found to impact on hyphal morphogenesis such as the exocyst complex and polarisome components as well as the Wiskott–Aldrich Syndrome-like gene *WAL1* [34,35,36]. Hyphal filaments in *Ashbya* are compartmentalized by septation. Septation is directed via landmark proteins and requires Cyk1-dependent actin ring formation as well as septin proteins [37,38,39,40,41,42,43]. Spindle pole body composition, the organization of cytoplasmic microtubules, nuclear migration and the distribution and asynchronous divisions of *Ashbya* nuclei in a shared cytoplasm have been intensely studied and revealed insight transferable also to other multicellular systems [44,45,46,47,48,49,50]. 

In this review, we describe research elucidating the molecular genetics of sporulation in *A. gossypii* and summarize open questions of fundamental fungal biology requiring further research.

## 2. Life Cycle of *Ashbya gossypii*

The life cycle of *Ashbya* shows morphological similarities with other filamentous fungi in its early stages, but peculiarities in its later stages. Spore germination begins with swelling of spores and the generation of a round germ cell [51]. In *Ashbya* germ cell formation occurs independent of the polarity establishment protein Cdc42 [25]. However, Cdc42 is essential for establishing asymmetry and the formation of a germ tube. Persistent localization of polarity proteins at the newly generated hyphal tip ensure its continuous polarized growth [26]. Characteristically, *Ashbya* germ cells form a second germ tube on the opposite side of the first germ tube. This feature of germ tubes positioned at a 180° angle is termed the bipolar germination pattern. Septation occurs at the base of these germ tubes and within the hyphal tubes. Sites of septation are predefined by positioning actin rings along the hyphae [37]. Septal sites also occur at the base of lateral branches and are directed by positional landmarks such as Bud3 [38]. These events regularly occur also in other filamentous fungi, e.g., *Aspergillus nidulans* [52].

**Figure 1:**
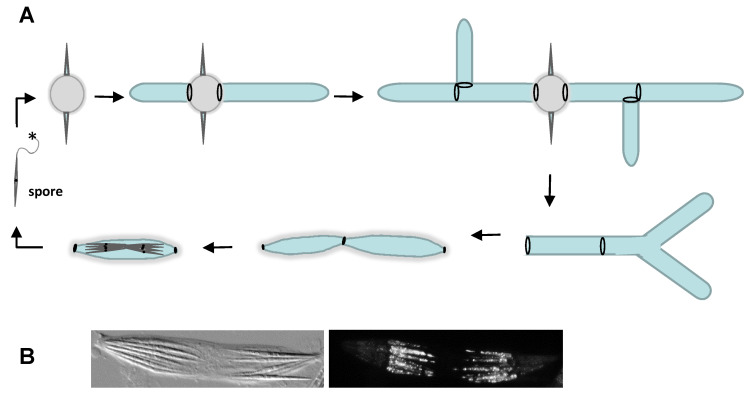
Life cycle of *Ashbya gossypii*. (**A**) A single spore (on the left depicted with its whip-like appendage marked by an asterisk) germinates, swells and forms a germ cell by growing isotropically. Then polarity establishment sets in and bipolar germ tubes are formed. A young mycelium grows and forms lateral branches. Upon maturation tips start bifurcating and at the end of the growth phase, e.g., in the interior of a colony, hyphae develop into sporangia, fragment and generate endospores. The ascus disintegrates and spores are set free. Spores form bundles by attaching via terminal whip-like appendages. *Ashbya* is a homothallic fungus, i.e., a single spore can form a sporulating mycelium. (**B**) Bright field (left) and fluorescent (right) microscopy images of a sporangium. The ascus was fixed with formaldehyde and stained with rhodamine phalloidin (see text for details). Scale bar is 10 µm.

Young mycelia of newly germinated *Ashbya* spores show a relatively slow growth speed, which increases considerably, i.e., > tenfold to about 200 µm/h, in mature hyphae [35]. About 24 h after induction of spores into rich medium, hyphal tips start a tip splitting process (Figure 1A). This replaces lateral branching for the formation of new hyphae and is characteristic for filamentous fungi in the genus *Eremothecium*. Septation not only compartmentalizes hyphae; hyphal segments are required for the formation of sporangia. Mutants that are aseptate, e.g., *cyk1*, fail to form sporangia and fail to sporulate [37]. Hyphal fragmentation during sporulation results in isolated sporangia that often bear eight spores. *Ashbya* spores harbor filaments at their tips and bundles of spores aggregate via these filaments. In a sporangium, two bundles of spores are formed connected via these filaments (Figure 1B). 

The shape of *Ashbya* spores is characteristic for the genus. *Ashbya* forms needle-shaped spores of about 30 µm in length. In contrast, *E. cymbalariae* spores are only half this size, lack the apical filaments and thus do not form bundles but occur as single spores [22]. *Ashbya* spores have two compartments. Only one is filled with cytoplasm and shows actin patches while the spore compartment distal to the filament is apparently solid and chitin rich (Figure 1B). Genes involved in polarized cell morphogenesis were found to also be involved in determining spore length via regulating the actin cytoskeleton [53].

Spore shape may have been selected by the insect vectors that inject these spores through their proboscides into plants. Interestingly, *Ashbya* is a very abundant sporulator. In minimal mediums, mycelia will be rapidly and quantitatively converted into sporangia, which then fragment and ultimately release their spores. Enzymatic breakdown of the sporangium wall may result in release of the spores. Of the two endoglucanases tested, *eng1* did not reveal a mutant phenotype while in the *eng2* mutant hyphal breakdown into single celled sporangia was abolished as well as spore wall breakdown [54]. 

This indicates that a single spore can complete this life cycle and generate a sporulating mycelium, i.e., *Ashbya* is a homothallic fungus. How homothallism is brought about is as yet unknown. Our current knowledge will be explored in the following sections and the outlook describes the need for further research in this area of fundamental fungal biology.

## 3. Developmental Programs Leading to Sporangium Formation

As stated in the previous chapter, septation is a prerequisite for sporulation. Septation occurs in regular intervals within hyphae. A cell compartment defined by septa will develop into a sporangium. The developmental pathway and its genes required for sporangium formation, however, remain to be elucidated. 

**Figure 2:**
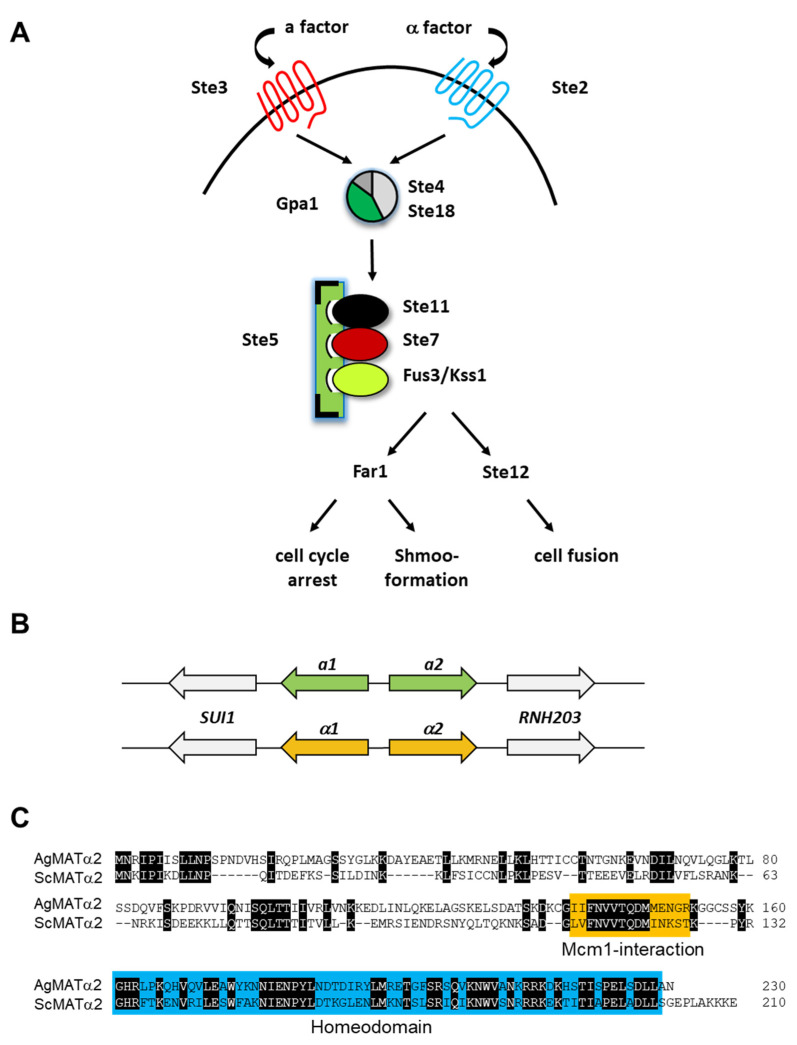
Mating type and pheromone signal transduction cascade. (**A**) The pheromone signal transduction cascade and its *Ashbya* genes based on *S. cerevisiae*. In *S. cerevisiae* there are three outputs of the cascade resulting in fusion of cells of opposite mating type and karyogamy to form diploid zygotes. The output of this cascade in *Ashbya* remains to be elucidated as mating has not been observed. For phenotypic characterization of mutant strains, refer to Table 1. (**B**) *MAT*-loci of *Ashbya gossypii* showing either *MAT**a*** or *MATα* with the highlighted mating type genes **a**1, **a**2 and *α*1 and *α*2 and adjacent genes. (**C**) Overexpression of *S. cerevisiae α*2 blocks sporulation in *Ashbya*. A protein alignment of *Ashbya* and *S. cerevisiae α*2 is shown marking conserved amino acids (shaded in black), the Mcm1-interacting domain and the C-terminal homeodomain.

In other fungal systems, sporulation is preceded by a pheromone-mediated attraction of cells of opposite mating type, cell fusion and then nuclear fusion to establish a diploid zygote. Diploidization may be directly followed by meiosis and sporulation or diploid stages may persist until adverse conditions initiate a sporulation protocol [55,56]. The homologs of the *S. cerevisiae* pheromone response signal transduction cascade are conserved in *Ashbya* (Figure 2A). However, key components can be deleted without inhibiting sporulation. 

Quite on the contrary, deletion of *STE12*, for example, actually generated a hypersporulating phenotype (see Table 1 for mutants and their phenotypes). In line with this, the type strain (ATCC10895) has not been observed to undergo cell fusion prior to sporulation (see also below) [57].

*Ashbya* is a homothallic strain as a single spore can generate a sporulating mycelium. There is no additional cell type required. The *Ashbya* genome harbors several mating type cassettes. Presumed inactive loci are located at telomeric ends of different chromosomes, while one locus, flanked by genes also found at other ascomycetous mating type loci, may harbor the active *MAT* locus. The ATCC10895 type strain, actually, only harbors *MAT**a*** loci, with ***a****1* and ***a****2* genes, whereas wild isolates were found possessing both *MAT**a*** and *MATα* (with *α1* and *α2* genes) information (Figure 2B) [19,21]. Yet, the type strain is sporulation competent. This poses a conundrum: how is this homothallism established solely based on a *MAT**a*** strain? In *Cryptococcus neoformans MATα* strains may undergo monokaryotic fruiting, that is they complete a full sexual cycle without the opposite mating partner [61,62]. This suggests that *Ashbya* could have evolved to perform haploid fruiting, e.g., in harsh environments found in the insect. This could have been for the sake of efficiency: a harsh environment could be fatal as the time wasted for finding a mating partner, undergoing mating and only then initiating sporulation could be better used to directly initiate sporulation. In molecular terms this relieves of the burden of mating pheromone interactions for finding a suitable partner— and potentially mating type switching to produce compatible cell types—zygote formation and the generation of an a1/α2 heterodimer. A multinuclear cell could thus provide for haploid nuclei to be able to fuse. However, this has not been studied so far. 

Since the type strain is solo-*MAT**a*** it the question arises if mating type has actually any role in sporulation? Interestingly, it was found that overexpression of *MATα2* in *Ashbya* blocks sporangium development and sporulation [58]. Due to the conservation of the general nature of the *α2* repressor between *S. cerevisiae* and *Ashbya*, a block of sporulation in *Ashbya* can be achieved with the *S. cerevisiae α2* gene (Figure 2C) [63]. Genes regulated by *α2* will be discussed below.

In *S. cerevisiae* Spo11 generates double-strand breaks inducing meiotic recombination [64,65]. *SPO11* mutants in *S. cerevisiae* decrease sporulation, but *SPO11* is apparently dispensable for sporulation in *Ashbya*. Dmc1 on the other hand, is required for repair of Spo11 induced meiotic DSBs in *S. cerevisiae* [66]. In *Ashbya dmc1* mutants are very poor sporulators, suggesting that in this strain nuclei that acquire a Spo11-DSB fail to repair this insult and thus fail to form spores. However, spores that are generated are healthy, germinate and form mycelia in a wildtype-like manner [54]. This finding may suggest that meiotic recombination may actually take place in *Ashbya* nevertheless (see below).

## 4. The Gear-Box of Sporulation

Based on a candidate gene approach, key homologs involved in sporulation in *S. cerevisiae* were studied in *Ashbya* [54]. This revealed that *IME1*, *IME2*, *IME4* and *KAR4* regulate a core set of genes that are activated by the transcription factor Ndt80. The *Ashbya* Ndt80 has a conserved DNA-binding consensus motif (RMCACAAAA) like ScNdt80p. Thus, these genes and the Ndt80 regulon represent the central core (gear box) of conserved sporulation genes. Specific for *Ashbya* is the hypersporulation phenotype of the *ste12* mutant. Deletion of *STE12* results in an approximately 10-fold overexpression of these gear box genes.

Global RNAseq transcriptomics were performed to obtain an overview of the sporulation gene set in *Ashbya* and to compare this set with *S. cerevisiae*. As expected, the developmental shift from growth to sporulation affects a large portion of the ~5000 *Ashbya* genes (protein coding and non-coding RNA genes) [67]. In total, 560 genes were found to be upregulated and some 300 downregulated. The downregulated genes are involved in translation and gene expression, e.g., of biosynthetic genes. Of the upregulated genes identified in *Ashbya* ~25% are also upregulated during sporulation in *S. cerevisiae*, suggesting core functions of sporulation but also a large degree of plasticity in achieving spore formation in different systems [54,59,68].

**Figure 3:**
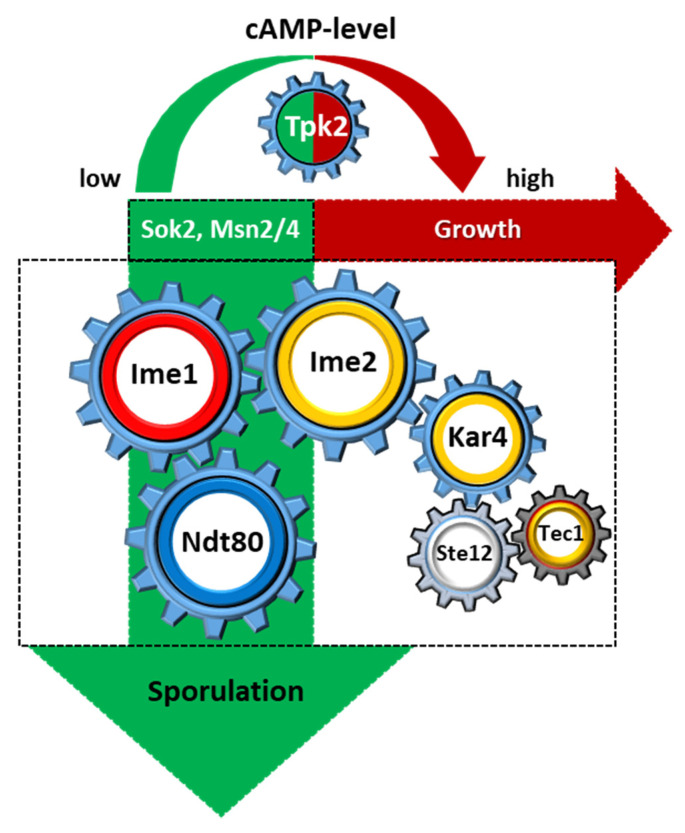
The Gear Box of *Ashbya* sporulation. Graphic description of the key regulatory components governing sporulation and the core set of 124 conserved sporulation specific genes. The cAMP/PKA pathway drives either vegetative growth or promotes sporulation depending on the nutrient status. High levels of alpha2 protein can lock cells in the vegetative state. For phenotypic description of mutant strains see Table 1.

The 124 conserved sporulation genes between *Ashbya* and *S. cerevisiae* encompass the core genes of sporulation and thus make up the gear box, whose central regulators are indicated in Figure 3.

We identified key intrinsic negative regulators of sporulation in *Ashbya* with the α2 repressor and *STE12*. A global comparison of the gene sets regulated by both factors has not been performed. However, α2 overexpression downregulates more than 300 genes of the *Ashbya* gene set upregulated during sporulation. This suggests that both α2 and Ste12 (see above) interfere with the gear box of sporulation itself (Figure 3). Ste12 functions downstream of the pheromone signal transduction cascade and consequently other mutants in genes of this cascade, including *ste7* and *ste11*, also result in an increase in sporulation. Ste12p may act in concert with Tec1 as deletion of *TEC1* itself also increases sporulation (Table 1) [54,57,60]. Expression of *IME2* was significantly upregulated in a *ste12* mutant and significantly downregulated by α2 overexpression, which suggests that α2 and Ste12 are gear sticks for *IME2*. RT-PCR indicated that *IME2* is not expressed in *Ashbya* under nutrient rich conditions, which was also observed in *S. cerevisiae* [54,69,70]. While *IME1* is weakly expressed in *Ashbya* even in nutrient rich conditions, overexpression of *IME1* is detrimental and results in aberrant growth. Interestingly, we found that a fungal specific transcription factor, encoded by the APSES protein Sok2, is downregulated in an α2 overexpressing strain. *SOK2* deletion completely blocks sporulation (although not sporangium formation), e.g., via downregulation of *IME2* [58]. Thus, the effect of α2 overexpression may be indirect.

The general nature of sporulation in a multinucleate cellular compartment is not understood. *Ashbya* cell compartments harbor several nuclei. Which of these nuclei contribute to spore formation and if some are, for example, degraded is unknown. The role of the spindle pole body in spore formation warrants further analysis [71,72]. Genetic evidence on the molecular processes during sporulation centers on the karyogamy genes *KAR3* and *KAR4* as well as the *DMC1* and *SPO11* genes. In *S. cerevisiae*, Kar4 is a transcription factor that activates transcription of the microtubule minus end directed kinesin motor protein Kar3. Kar3 provides the force for nuclear congression during mating [73,74]. *Ashbya* mutants in *KAR3* and *KAR4* show only mild growth defects, yet strong sporulation defects. This suggests a potential role of karyogamy for sporulation in *Ashbya* [54]. 

## 5. Environmental Control of Sporulation

In *S. cerevisiae*, *IME1* is viewed as the central regulator of meiosis. The activation of this gene is controlled by multiple pathways that converge on the long promoter of *IME1*. Part of this regulation is transcription of a long non-coding RNA, lncRNA *IRT1,* through the promoter of IME1 [75]. Additionally, there is a second non-coding RNA, *IRT2*, which is part of a positive feedback loop to stimulate *IME1* expression [76]. 

In *Ashbya* the *IME1* promoter is rather small, but it contains putative consensus sequences for Tec1, Mcm1 and Ndt80 (based on the *S. cerevisiae* consensus sequences for Tec1 and Mcm1-binding and the Ndt80 consensus also defined in *Ashbya*; http://www.yeastract.com/).

*Ashbya* is a very potent sporulator. We hypothesize that living in a harsh environment requires some survival skills and sporulation may be the best option to survive association with an insect vector. In general, fungi do not sporulate when ample nutrient supplies are available. The currency that monitors nutrient status in the cell is cyclic AMP [77]. Hence, mutations interfering with cAMP/PKA signaling affect sporulation. In *S. cerevisiae* deletion of *BCY1*, encoding the regular subunit of PKA abolishes sporulation [78]. Similarly, overexpression of the catalytic subunit of PKA, pkaC1, in *Aspergillus fumigatus* severely reduced sporulation efficiency [79]. Addition of exogenous cAMP blocks sporulation in *Ashbya* and downregulates most of the sporulation specific genes [59].

In *Ashbya,* there are two catalytic subunits of PKA, Tpk1 and Tpk2. Deletion of cPKA genes showed that *tpk1* strains still responded to nutrient cues and blocked sporulation when challenged with exogenous cAMP, while *tpk2* strains were blind to high levels of cAMP and initiated sporulation. Since Sok2 is a potential downstream target of PKA it places this transcription regulator in a central position to integrate different signaling pathways into a developmental response [80,81].

## 6. Spore Wall

A dityrosine layer is found in *S. cerevisiae* to protect its spores [82]. Homologs of the *DIT1* and *DIT2* genes were found in *Ashbya*. During sporulation *DIT1* and *DIT2* are dependent on the transcriptional regulator Msn2/4. *MSN2/4* is regulated by PKA in *S. cerevisiae. MSN2/4* deletion in *Ashbya* leads to a reduction in spore formation and a severe loss of viability/germination efficiency of these spores. Several genes involved in spore wall formation were found to be downregulated in an *msn2/4* strain, which could explain the poor spore viability observed [59].

Formation of spindle-shaped *Ashbya* spores requires the actin cytoskeleton and its regulatory proteins. Recent research elucidated the role of the formin Bnr2 in sporulation. Interestingly, it was found that Bnr2 resides in the outer plaque of the spindle pole body to mediate actin assembly. The spindle pole body (SPB) is embedded in the nuclear membrane. The outer component of the SPB, the outer plaque, anchors the γ-tubulin complex but during meiosis recruits sporulation specific components [72]. Deletion of another component of the outer plaque, encoded by *MPC54*, generated elongated spores that showed defects in spore wall formation [71]. 

*Eremothecium* spores contain 3-OH oxylipins (oxidized fatty acids), which may be involved in spore release, aggregation and dispersal [83,84]. These compounds are not specific to the genus *Eremothecium* but can be found in other ascomycetous genera, and also in the distantly related genus *Saccharomycopsis* [85].

## 7. Do Not Miss the Wake-up Call: Signals for Germination

Spore germination, i.e., the breaking of spore dormancy, requires the presence of water and nutrients, particularly glucose in *S. cerevisiae* [86]. Then a concert of transcription factors, including Sok2, orchestrates germination subprograms, [87]. In *Schizosaccharmyces pombe* signaling via the cAMP/PKA-pathway is required for spore germination and mutants in the catalytic subunit of PKA, *pka1*, showed severe germination defects [88]. Similarly, we found that *Ashbya* mutants in *TPK2* showed strong germination defects [59]. This indicates that Tpk2 is required as a developmental regulator both at the entry of sporulation and at the exit of spore dormancy (Figure 3). 

## 8. Outlook: Open Research Questions and Unknown Territory

Molecular genetic work over the last 30 years on *Ashbya* has made some fundamental contributions to yeast research. The most well-known may be the proof of the whole genome duplication in *S. cerevisiae* [19,20]. The molecular analysis of riboflavin production and the novel bioengineering efforts to implement *Ashbya* as a platform strain for proteins, lipids or flavor compounds has made *Ashbya* a very interesting strain for the biotech industry as an alternative and addition to *S. cerevisiae* and other yeasts as well as for synthetic biology approaches [3,6,7,9,10,18]. Developmental biology contributed to the analysis of polarized hyphal growth, septation, nuclear migration and asynchronous nuclear divisions in a filamentous fungus [22,23,24,27,44,45,46,47,48,49]. Here *Ashbya* has proven a valuable system for comparative biology studies. Due to its small genome, studies on genome evolution could trace back the ancestral genome of the *Eremothecium* lineage [23]. This could aid deciphering the molecular events leading to filamentous growth, multiple nuclei per cell and its life cycle. 

Recent work, particularly combining gene function analyses and RNAseq transcriptomics have generated substantial insight into the sporulation process in *Ashbya*. Core similarities with *S. cerevisiae* were uncovered encompassing a gear box of sporulation functions and the role of nutrient signaling via the cAMP/PKA pathway as well as specific differences revealing distinct regulatory processes in *Ashbya*. However, there is a large number of open questions concerning the general lifestyle of *Ashbya* and the detailed regulatory events, i.e., the occurrence of meiotic divisions during sporulation. Furthermore, general fungal specific questions on the genetic program of spore germination and the breaking of spore dormancy remain to be answered. Key future areas of *Ashbya* research on sporulation are these:

(i) How is homothallism and haploid fruiting regulated in *Ashbya*? There is a lack of evidence for mating, cell fusion and karyogamy in *Ashbya*. Most of the research is performed with the type strain ATCC10895. Other isolates should be studied in comparison. Interestingly, there is an older report that indicates formation of a secondary mycelium that is generated by germinating spores that then undergo fusion and produce secondary spores of different shape [1]. Essentially, this describes a similar phenomenon of what became known as CATs (conidial anastomosis tubes) in *Neurospora crassa* [89,90,91]. This needs to be reinvestigated and studied on the molecular level.
(ii) *KAR4* and *STE12* mutants have quite opposite phenotypes in *Ashbya*. It is not understood how these transcriptional activators differentially regulate the same gene set. In *S. cerevisiae*, Ste12 and Kar4 may co-regulate specific genes [92]. One possibility is that deletion of *STE12* enables Kar4 to better access sporulation gene promoters explaining the hypersporulation phenotype, while deletion of *KAR4* abolishes sporulation as Ste12 alone cannot activate gear box genes. This could be analyzed by *KAR4/STE12* overexpression studies.
  Ste12 is at the bottom of the pheromone-response signal transduction cascade [93]. The role of this cascade for *Ashbya* biology remains to be elucidated. One hypothesis is that there may be autocrine signaling in *Ashbya* by which its own pheromone production regulates filamentous growth and the developmental switch to sporulation, particularly as we lack evidence of mating interactions in *Ashbya*. Autocrine pheromone signaling has been observed in the distantly related fungi *C. neoformans* and *Ustilago maydis* [94,95]. Attempts to overactivate the pheromone signal transduction cascade using an AgSte7-DD allele, in which residues potentially regulated by phosphorylation were mutated into glutamate to mimic activation (according to [96]) did not result in an altered sporulation behavior (our unpublished results).
(iii) The developmental cascade leading to cellular growth resulting in sporangium formation and the genes involved are presently unknown. A role of pH-regulation in sporulation remains to be studied. Several of the gear box mutants tested are still able to form sporangia. Interestingly, return-to-growth studies indicated that sporulation mutants can generate new hyphal growth and return to mitotic divisions even after several days in sporulation medium.
(iv) By RNAseq transcriptomics several unique *Ashbya* genes highly induced during sporulation have been identified. Their role is unknown and remains to be elucidated.
(v) Breaking the dormancy of spores is a topic of general interest in fungal biology. Nutrients and the cAMP/PKA pathway certainly play a role. Our data clearly link spore germination to the catalytic PKA subunit encoded by *TPK2*. However, the downstream targets are unknown. They could be identified using phospho-proteomics to identify proteins specifically phosphorylated during germination. Sok2 may be a prime candidate. However, since *sok2* mutants cannot sporulate, a potential role of Sok2 in germination has not been studied yet. Conditional expression of *SOK2* in spores, e.g., using regulatable promoters could help to elucidate its role in germination.
  In other systems there is a quorum sensing mechanism in spores. This mechanism informs a spore if there are other siblings around that have already started to germinate or grow. This ensures that a spore does not miss an opportunity to grow and is enticed to wake up as well. In *Bacillus subtilis*, muropeptide cell wall fragments released from germinating spores activate a *prkC* encoded Ser/Thr kinase receptor that, for example activates EF-G by phosphorylation [97]. Similar mechanisms have not been identified in fungi so far. Spore germination experiments with *Ashbya* suggest that the number of germinating spores (i.e., the quorum) has an influence on cell morphology of young germlings. Glycopeptide release from germinating spores of fungal plant pathogens may act as elicitors of plant defense responses, e.g., as shown for *Mycosphaerella* [98]. These molecules could therefore also function as microbe-associated molecular patterns triggering plant immunity [99,100].

This list shows that there is still uncharted territory to map out in fungal sporulation and spore germination in the future.

## Figures and Tables

**Table 1:** Gene-function analyses with respect to sporulation behavior.

Gene	Phenotype	Genetic Alteration	Reference
*ScMATα2*	no sporulation	overexpression	[58]
*ime1*	no sporulation	deletion	[54]
*ime2*	no sporulation	deletion	[54]
*ime4*	no sporulation	deletion	[54]
*kar4*	no sporulation	deletion	[54]
*ndt80*	no sporulation	deletion	[54]
*dig1*	no sporulation	deletion	[54]
*sok2*	no sporulation	deletion	[58]
+cAMP in WT	no sporulation	none, exogenous addition of cAMP	[59]
+cAMP in *tpk1*	no sporulation	none, exogenous addition of cAMP	[59]
*dmc1*	severely reduced sporulation	deletion	[54]
*kar3*	severely reduced sporulation	deletion	[54]
*spo14*	severely reduced sporulation	deletion	[54]
*ume6*	severely reduced sporulation	deletion	[54]
*spo1*	reduced sporulation	deletion	[54]
*msn2/4*	poor spore viability	deletion	[59]
*ste2/ste3*	sporulation	single or double deletion	[57]
*spo11*	sporulation	deletion	[54]
+cAMP in *tpk2*	sporulation	deletion, exogenous addition of cAMP	[59]
*tpk1*	sporulation	deletion	[59]
*tpk2*	sporulation	deletion	[59]
*ste11*	increased sporulation	deletion	[54]
*ste7*	increased sporulation	deletion	[54]
*ste12*	increased sporulation	deletion	[57]
*tec1*	increased sporulation	deletion	[60]

Note: background color identifies differential sporulation competence.
